# Annotations

**Published:** 1890-03-08

**Authors:** 


### Annotations.

**Alcohol in Hospitals.**

There are very few persons who do not hear with pleasure of diminution in the consumption of alcohol. As foods, wines, spirits, and beers are seldom needed. Those who are injured by them are out of all proportion in numbers to those who are benefitted. The proper places for most alcoholic substances are the chemist's shop and the doctor's dispensary. If alcohol were chiefly used as medicine and under medical instructions it is probable that temperance societies would cease to exist. In the meantime, however, the British public by no means either appreciates the value of such societies or treats them with the gratitude they deserve. A few days ago the British Medical Temperance Association met under the presidency of Dr. B. W. Richardson. A report was presented, signed by Dr. Thomas Morton, Dr. Walter Pearce and Mr. John Moir, in which a comparison was made between the average expenditure of hospitals on alcohol twenty-five years ago and at the present date. Seventy-three hospitals were included in the report; and it was shown that the quantity of alcohol administered to patients at those hospitals to-day is little more than half of what it was a quarter of a century ago. At some of the institutions the comparative figures are far more striking than these. King's College Hospital for example, which spent £7 14s. 1d. per bed per annum on alcohol in 1863, only spent £1 8s. 8d. in 1888; whilst the Sunderland Infirmary, which spent no more than £1 7s. 6d. in 1863, had reduced its expenditure on this head to 4s. 4d. in the latter year. More gratifying still is the fact that the London Fever Hospital, which used to spend in alcohol £3 5s. per bed per annum, now spends only 15s. 9d. From every point of view, except perhaps that of the brewer and the wine merchant, this change is in the highest degree satisfactory. Whilst we avoid the fanaticism of the position that alcohol can never under any circumstances be of service to human beings, we maintain, on the other hand, that temperance, amounting to practical abstinence, is the best rule of life both for the body and the mind, both for the sick and the well. The Temperance Hospital has rendered admirable service alike to science and to man by its persistent faith in non-alcoholic treatment. Dr. Morton and his colleagues have done well in that they have collected and published such valuable statistics on so important a practical question.

**The Lady and the Doctor.**

Dr. Herbert Cooper was undoubtedly justified in bringing Mrs. A. E. Somerville before the magistrates the other day for striking him on the face with a whip. The "flagellum," it is true, was but a toy whip, and Mrs. Somerville considered she had a grievance against the doctor for having signed a lunacy certificate whereby she was consigned to Camberwell House Asylum. It appears that she had previously brought an action for libel against Dr. Cooper in reference to the certificate, and that the case had been dismissed in October last with full costs on the higher scale. The lady, however, still dissatisfied, went to Dr. Cooper's house a short time ago, provided with a whip, and evidently determined to take the law into her own hands. She thus became defendant instead of plaintiff in a new action. But this time also the case was decided against her, and again with costs. We sincerely sympathise with persons so unhappily afflicted as Mrs. Somerville seems to have been. It is manifest that the Lord Chief Justice considered the doctors who certified her lunacy to have acted honourably and according to the evidence. The whole circumstances, indeed, go to show that Dr. Cooper performed a painful professional duty with kindness, impartiality, and competent skill. The after history of the case, taken in conjunction with many similar histories, raises the important question whether there should not be some change made in the law, whereby medical men may be adequately protected in the discharge of their onerous avocations. It cannot be, it never is, other than a painful thing for an honourable medical man to deprive a fellow creature of liberty by means of a lunacy certificate. It is no more profitable than pleasant, for the whole fee attaching to the giving of such a certificate is usually no more than a guinea. Is it possible to suppose that any man, however poor, will help to consign another to a lunatic asylum for the miserable reward of a guinea? No doubt, in the course of the last hundred years a very few medical men have lent themselves to conspiracies whereby sane persons have been immured within the walls of what have proved life-long prisons. Such things are so horrible in themselves and so horrifying to the general imagination that they have been laid hold of by sensational novelists, and worked up into Dantean pictures, upon which all the world has gazed with mingled terror and rage. There are few persons of middle life who have not pictured themselves the victims of foul conspiracy and the life-long tenants of a madhouse. But in all this a mere ounce of fact has been the parent of whole universes of fiction. The result is that nowadays it is not persons wrongly certified as lunatics who are the victims, but medical men who give honest lunacy certificates. The law as it stands gives practically no protection to a doctor in the exercise of one of his most painful and responsible duties. There really is no reason why doctors alone should be selected to certify lunatics. Any man of reasonable common sense is as well able to diagnose a case of lunacy as the average physician. If doctors were really men of business and possessed of that saving common sense which the keen competition of the world compels business men to exercise every day, they would promptly throw overboard the responsibility for lunatics which mistaken legislation has imposed upon them. One of two things will certainly happen: either medical men must be adequately protected in the discharge of their duties, or the Legislature must find new methods of dealing with all classes of lunatics.

**Monkeys and "Tight Lacing."**

It has for some time been manifest to impartial observers that scientific knowledge does not necessarily co-exist with good sense and good feeling in the same person. Good sense and good feeling are, in fact, a little at a discount with some scientific men. But those are mostly young men, who, it may be hoped, will know better by the time they have become mentally mature. Not always, however, is it that those scientists who seem to lack both sense and good feeling are young. Dr. Lauder Brunton, for example, is not young, but of ripe age. Yet it is to Dr. Brunton that we owe the peculiar experiment of "tight lacing," or rather encasing in plaster of Paris, a monkey or monkeys. What fact of scientific importance did Dr. Brunton hope to demonstrate which has not been already demonstrated over and over again in the human subject? Had not the hundreds of animals he had already experimented upon proved all he was in search of? Is there a single newspaper reader anywhere who does not know that tight-lacing compresses the lungs and the heart, the stomach and the bowels, the liver and the spleen, and practically, every other organ both in the abdomen and the chest? The consequences of such compression are so universally familiar that if but a girl faint in a ball-room the most inexperienced among those present will rush to unfasten her corsets. Is there any conceivable use in re-demonstrating things that are as well-known as the multiplication table, or the price of a glass of bitter beer? A man owes something to his years, his position, and the profession of which he is a member. No physician can make such trivial and inconsequent experiments as Dr. Brunton reports himself to have made without bringing himself and the whole medical profession into something like contempt. Those persons who have written to the daily papers seem surprised even to astonishment that a grave physician of mature years should behave in so childish and irresponsible a fashion. And well they may! Experiments on animals have undoubtedly been one of the chief means of making a science of physiology possible. But such experiments can never be looked upon as anything but grievous and painful necessities. No man should undertake them except on the most clear conviction that he is about to acquire knowledge which cannot be acquired in any other way, and to demonstrate facts the knowledge of which will be of service to man for all time. To needlessly multiply such experiments from a mere whim of curiosity, or with a desire to spread abroad his own fame, is the work of a devil and not of a man. If these experimenters have not forgotten all they ever learnt in their youth, they must have many a qualm of conscience in their better moments.

